# Supplementary material for: Human knowledge models: Learning applied knowledge from the data
Source: PLoS One. 2022 Oct 20;17(10):e0275814. doi: 10.1371/journal.pone.0275814 (PMC9584406; doi:10.1371/journal.pone.0275814)
Supplement: S1 File — (DOCX) [file pone.0275814.s001.docx]

# Data availability

The data for E-, H-, and F- sets used in this study are available from standard public repositories (<https://www.kaggle.com/adityakadiwal/water-potability>, <https://www.kaggle.com/datasets/S%C3%ADrio-Libanes/covid19>, and <https://archive.ics.uci.edu/ml/datasets/Polish+companies+bankruptcy+data#> respectively; see [39], [38], and [40]). The dataset for S-set, that we have created ourselves, is provided with this paper as Supplemental material.
