# Supplementary material for: Human knowledge models: Learning applied knowledge from the data
Source: PLoS One. 2022 Oct 20;17(10):e0275814. doi: 10.1371/journal.pone.0275814 (PMC9584406; doi:10.1371/journal.pone.0275814)
Supplement: Attachment — Submitted filename: Answers to the Reviewers.ver11.docx [file pone.0275814.s004.docx]

PONE-D-22-19468

Human Knowledge Models: Learning Applied Knowledge from the Data

PLOS ONE

Answers to the reviewers

We would like to thank all the reviewers for their constructive feedback. We were able to incorporate all suggestions into the revised paper, and our detailed answers are provided in this document.

# I. Reviewer #1

In this paper the authors introduce the class of Human Knowledge Models (HKMs), as an attempt to formalize the set of functions that humans can efficiently compute without any additional resources. The contributions are novel and the organization is good.
However, the following minor corrections to be done before consider this paper for publications.

Answer:

Thank you for your constructive feedback. Your suggestions were incorporated into the new version of the paper, and we provide our detailed answers to your comments below.

1. The Introduction is small, and it do not clearly explain the motivation. It is recommended to provide the challenges of the existing models and motivation clearly.

Answer:

We tried to keep the introduction concise to save more space for the main part of the paper (where we had to cover several important aspects of HKMs). We list several barriers to the use of most current ML models in the introduction to establish our motivation, and we show later in the paper how HKMs overcome these barriers.

2. The references considered for this work are older. Consider the state-of-the-art papers for references. Some of the references are available in the existing papers.

Answer:

We added several more current references most related to our work, as suggested.

3. The results are well presented, but the reasons for achieving the better performance of proposed work over the existing approaches to be discussed.

Answer:

Thank you for your feedback. We added more information about possible reasons.

The potential reasons for the HKMs surpassing black boxes models are provided in Section 3.1. In short, they can be attributed to the greediness of existing rule-based models and to the “natural complexity” of the dataset. In order to run in a reasonable time, existing rule-based models exploit various empirics and greedy algorithms, often leading to suboptimal solutions. Exhaustive search, which becomes affordable for the small-size HKM models, will produce better results.

As for the data complexity, features (columns) in real-world datasets vary not only by their number, but also by their expressive power. It is possible that four well-constructed features of the dataset will explain the most deviation in the target label, while hundreds of other features will only provide a few adjustments. So a complex black box model might not be required, if a few best features capture the essence of the process.

# II. Reviewer #2

The authors present an interesting manuscript on human knowledge models in AI. Study design and research questions are clearly described. In this sense, it is easy to understand the aim of this study. The bright side of the manuscript is that to provide some useful practical details on related topic. In this context, the study contributes to different fields. The authors explained their methods, and results but some sentences of the paper manuscript (highlighted the pdf file) are too long and there are some grammatical mistakes. Therefore, I would like to make some minor suggestions to improve the quality of the paper as below:
In general, methods and results should be written in past tense but some in some parts of the manuscript some sentences were written in simple present tense.
In methods and results sections, authors used “As a result” in several times (highlighted the pdf file) and this disrupts the flow of the subject and the continuity of the reading. Instead of this using this term, authors should clearly refer the related objective/results or method.
Manuscript section names and reference styles should be corrected by the journal guidelines.

Answer:

Thank you for all of this helpful feedback. We have incorporated your suggestions below, as well as those you highlighted in the pdf.

Abstract
Abstract should be one paragraph, please unite two separate paragraphs.
we formalize -> we formalized
We use -> we used
Our results prove -> Our results proved

Answer:

Corrected as you suggested: past tense and single paragraph.

How the results contribute to further studies? A few words about to whom the results will be important will be valuable. In my opinion, it is always good to finish the abstract with such a sentence.

Answer:

Corrected as suggested (using our sentence from the Introduction, as you suggested below).

Introduction
-Authors defined the purpose of the work and its significance in introduction section. However, introduction should briefly place the study in a broad context and highlight why it is important. In this context, in my opinion, introduction should be started with a paragraph that defines the importance of AI/machine learning algorithms/models which benefits the various research fields by giving several examples from different fields (e.g. ecology, healthcare, software development etc.). Then, authors can continue with explaining the limitations of these AI/machine learning algorithms/models. In this way, bridge between the problem and objectives of the study would be stronger.
For example, Akçay et al. (2020), found that computer-aided counting outperformed the manual counting with respect to both accuracy and time (doi: 10.3390/ani10071207). There are many examples available from different fields.
In other words, authors should mention that “yes AI or machine learning models help/contribute researchers to archive their tasks and/or solve problems in less time but the models have limitations and need to be improved”.

Answer:

-The following sentence should not be in introduction section. Authors may add this sentence to abstract as a last sentence or results section.
“As a result, our study reveals the great potential of HKMs, particularly in the decision-making applications where “black box” models cannot be accepted. Moreover, this improves our understanding of how well human decision-making, modelled by HKMs, can approach the ideal solutions in real-life problems.”

Answer:

Agree, and we moved this sentence to become the last sentence of the Abstract (which also addressed your other comment above).

Conclusion
A few words about future work and to whom the results will be important will be valuable. Authors may say with a sentence that their findings contribute the different fields such as medicine, healthcare, ecology, software development etc…

Answer:

Corrected as you suggested, thank you.

Also, I made some highlights on the manuscript pdf file.

Answer:

Thank you for this detailed feedback, it is reflected in the updated version.

# III. Reviewer #3

The article proposes a new model (HKM). The work seems to be very interesting.

Answer:

Thank you for sharing our interest in this new field.

The following are the queries are from my side:
1. In what way, does HKM differ from Reinforcement learning?

Answer:

The principal difference between HKMs and any other AI field, Reinforcement learning included, is that HKMs are built to mimic human computational abilities. This significantly limits the choice of functions used in HKMs, but this makes HKM results look like organic human knowledge (as if it were discovered by the humans).
On a more detailed level, HKM is a form of supervised learning, not driven by rewards, as Reinforcement learning is. Moreover, HKM discovers both the best choice of features and functions, which the standard AI models usually optimize predefined functions (linear regressions, SVMs, neural nets, etc.).

2. What is the rationale behind choosing 4 features and how are these 4 features chosen?. The number of features varies across the datasets.

Answer:

The rationale for using up to 4 features comes from studies on human cognitive abilities, as indicated in the paper. In other words, this is the maximum number of features an average human is using in decision-making. HKMs are designed to mimic human-level computing (see our comment above), so this becomes one of the most principal criteria.

The number of all features is certainly varying across different datasets. But HKMs are used to find the best 4-feature model(s) from all the features provided originally. That is, the original dataset may have 10 or 100 features, but HKM will use only 4 of them, providing the best explanation of the outcome.

Finally, it is not only the number of features that varies across the datasets, but also the expressive power of these features in describing the target label. For example, the financial dataset (F-set) in the paper consists of 64 equally important features, while the healthcare dataset (H-set) consists of 229 features from which only a few are important. So, in practice, one might study the dataset and develop more and more expressive features. Then, even 4 features used in Human Knowledge Models would be enough to predict the target label.

3. if possible, provide a quantitative tool for measuring the performance of HKM?

Answer:

HKM performance was measured with standard metrics, such as F1, precision, and recall, as described in the paper. In this regard, HKM is not different from any other model. Moreover, using standard metrics helps compare HKMs to the other standard machine learning models.

4. even though the authors used the data as such to maintain the originclaity, will not have any impact on the performance?

Answer:

If you refer to the performance of the HKM exhaustive search, we prove that it is polynomial with respect to the number of features (see Equation 3 in the paper). That is, for the maximum of 4 features, HKM time complexity is proportional to the 4^th^ degree of the original feature count. This rather reasonable bound, combined with fast Boolean math used in HKMs, and parallelizable HKM search algorithm, leads to good performance of HKM training.

5. M best HKM models of n variables are produced as output? How do they compare to each other?

Answer:

You are correct: HKM search produces not one (as in standard AI), but several (M) best models. This is essential as each model often represents a different explanation of the reality (outcome). For example, patient examinations may take longer because patients are older, have disabilities, have certain risk factors, etc. In such cases, it is critical to know and understand all reasons which can explain the outcome.

The number M is usually kept small (such as 10 or 20), so that a human can look through all these best models and understand their “reasons”. Then, for small M, the top M models usually have very similar performance, but it also depends on the actual dataset. For example, if the dataset has only one feature strongly predictive of the outcome, then only the model(s) with this feature will make it to the top, and the other models will be ignored.

6. Whether the authors tested HKM models by random querying?

Answer:

All models and results used in our paper were computed with the same classical train/test approach – meaning that the data was randomly split into several folds, the models were trained on some, and tested on the others.

7. Whether the models generated for one dataset can be used for another? That, is it possible to transfer the knowledge gained? Or Update the knowledge when the dataset is added with new features.

Answer:

As our results demonstrate, HKM produced the most consistent results on cross-validation (randomized) training. This means that HKMs will actually be the most transferrable compared to the other standard AI models that we have considered. Moreover, since HKM logic is perfectly transparent to humans, they can be easily updated if needed, either by retraining, or by the human users directly – as you are suggesting.
